# *N*-Docosahexaenoylethanolamine Attenuates Neuroinflammation and Improves Hippocampal Neurogenesis in Rats with Sciatic Nerve Chronic Constriction Injury

**DOI:** 10.3390/md18100516

**Published:** 2020-10-15

**Authors:** Anna A. Tyrtyshnaia, Evgenia L. Egorova, Anna A. Starinets, Arina I. Ponomarenko, Ekaterina V. Ermolenko, Igor V. Manzhulo

**Affiliations:** A.V. Zhirmunsky National Scientific Center of Marine Biology, Far Eastern Branch, Russian Academy of Sciences, Palchevs.kogo Str, 17, 690041 Vladivostok, Russia; envelope2006@yandex.ru (E.L.E.); anan.star13@yandex.ru (A.A.S.); arina.ponomarenko.93@mail.ru (A.I.P.); ecrire_711@mail.ru (E.V.E.); i-manzhulo@bk.ru (I.V.M.)

**Keywords:** *N*-docosahexaenoylethanolamine, DHEA, synaptamide, hippocampus, neuropathic pain, microglia, chronic constriction injury, neurogenesis

## Abstract

Chronic neuropathic pain is a condition that causes both sensory disturbances and a variety of functional disorders, indicating the involvement of various brain structures in pain pathogenesis. One of the factors underlying chronic neuropathic pain is neuroinflammation, which is accompanied by microglial activation and pro-inflammatory factor release. *N*-docosahexaenoylethanolamine (DHEA, synaptamide) is an endocannabinoid-like metabolite synthesized endogenously from docosahexaenoic acid. Synaptamide exhibits anti-inflammatory activity and improves neurite outgrowth, neurogenesis, and synaptogenesis within the hippocampus. This study aims to evaluate the effects of synaptamide obtained by the chemical modification of DHA, extracted from the Far Eastern raw material *Berryteuthis magister* on neuroinflammatory response and hippocampal neurogenesis changes during neuropathic pain. The study of microglial protein and cytokine concentrations was performed using immunohistochemistry and ELISA. The brain lipid analysis was performed using the liquid chromatography-mass spectrometry technique. Behavioral experiments showed that synaptamide prevented neuropathic pain-associated sensory and behavioral changes, such as thermal allodynia, impaired locomotor activity, working and long-term memory, and increased anxiety. Synaptamide attenuated microglial activation, release of proinflammatory cytokines, and decrease in hippocampal neurogenesis. Lipid analysis revealed changes in the brain *N*-acylethanolamines composition and plasmalogen concentration after synaptamide administration. In conclusion, we show here that synaptamide may have potential for use in preventing or treating neuropathic cognitive pain and emotional effects.

## 1. Introduction

Neuropathic pain is a pathological condition associated with injury or disease of the somatosensory nervous system. Such damage of the nervous tissue can be both central and peripheral, thus creating various pain syndromes [1]. Peripheral neuropathic pain is a widespread and complex pathological condition that can have a variety of etiologies, including nerve trauma during surgery or compression neuropathies such as carpal tunnel syndrome, nerve compression, physical nerve trauma, and various plexopathies [2]. The prevalence of neuropathic pain syndrome in the population is influenced by various factors, including aging, diabetes, and cancer incidence increase, as well as the effects of chemotherapy that affect all sensory fibers. Such pain seriously impairs quality of life and imposes significant costs on society. Existing pharmacotherapy options, such as nonsteroidal anti-inflammatory drugs, antidepressants, anticonvulsants, and opiates predominantly relieve pain symptoms only through the nonspecific reduction of neuronal excitability [3]. These common therapies do not target the underlying causes of pain, and only rarely lead to the complete relief of symptoms. The ineffectiveness of the available treatments is mainly due to a limited understanding of the processes underlying this type of pain. Neuropathic pain is manifested through a combination of different sensory symptoms (allodynia, hyper- and hypoalgesia, hyper- and hypesthesia) as well as cognitive and affective disorders including memory loss, anxiety, depression, and anhedonia. These symptoms suggest the involvement of various brain regions, including the hippocampus [4,5] in the pathological process. Numerous studies indicate that chronic peripheral pain can cause profound changes in hippocampal plasticity, including impaired neurogenesis [6] and synaptic plasticity. Prolonged neuropathic pain can affect emotionally guided learning and associated synaptic reorganization, which contributes to the chronicity of pain syndrome. An integral part of the pathogenesis and the driving force of such reorganization is the reaction of neuroinflammation, accompanied by microglial activation and pro-inflammatory factor release. Glial cells activated in neuropathic pain are characterized by proliferation, hypertrophy, and increased production of proinflammatory cytokines, such as interleukin-1β, interleukin-6, and tumor necrosis factor-α [7,8,9].

**Figure 1:**
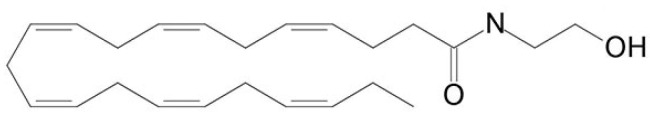
Chemical structure of *N*-docosahexaenoylethanolamine (DHEA, synaptamide).

An integrated approach to treatment involving the simultaneous use of anti-inflammatory and analgesic drugs may be the most effective to relieve pain and prevent the cognitive consequences of neuropathic pain. Recent evidence suggests that n-3 polyunsaturated fatty acids (PUFAs) exhibit anti-inflammatory activity through the synthesis of resolvins, protectins and maresins (bioactive mediators with pro-resolving properties). These mediators play a critical role in inflammation resolving [10]. In addition, PUFAs act on the cell membrane, providing a stabilizing effect and making it more resistant to damaging influences and more malleable for nerve impulse conduction [11]. Since the most important pathological factor of neuropathic pain is the neuroinflammation reaction within the spinal cord and the brain, the use of docosahexaenoic acid (DHA), which has anti-inflammatory properties, is very promising [12,13]. However, DHA is believed to be active primarily through its derivatives and metabolites. Moreover, the anti-inflammatory activity of omega-3-PUFA oxidative derivatives significantly exceeds the activity of precursors [14]. *N*-docosahexaenoylethanolamine (DHEA, synaptamide) (Figure 1) is a metabolite similar in structure to endocannabinoids, and is synthesized endogenously from DHA, an essential brain polyunsaturated fatty acid. Synaptamide promotes neurogenesis, neurite outgrowth and synaptogenesis in developing neurons, stimulating glutamatergic synaptic activity in hippocampal neurons [15,16] as well as attenuating neuroinflammatory response [17]. The term “synaptamide” was coined for DHEA to highlight its potent synaptogenic activity. Synaptamide is an integral participant in hippocampal neuron development and has a neuroprotective effect [16]. Progress in understanding the synaptamide effect on hippocampal-dependent functions in neuropathic pain will stimulate the development of new therapeutic strategies for the treatment of diseases caused by somatosensory nervous system damage. 

The present study aims to evaluate the effect of synaptamide on neuroinflammatory response within the hippocampus as well as hippocampal neurogenesis changes produced by neuropathic pain. We investigated the effect of synaptamide on neuropathic pain severity, hippocampal-dependent memory, microglia activity, and hippocampal neurogenesis in a rat CCI model. The animals used in the experiment were divided into four groups: (1) the “Sham” group, in which sham-operated rats were treated with the vehicle (water); (2) the “Sham + Syn” group, in which the sham-operated animals were treated with synaptamide; (3) The “CCI” group, in which the CCI animals were treated with the vehicle (water); and (4) the “CCI + Syn” group, in which the CCI rats were treated with synaptamide.

*N*-docosahexaenoylethanolamine was obtained by chemical modification of DHA extracted from Far Eastern raw material, namely, the processed products (digestive gland) of the commander squid *Berryteuthis magister* caught in the Bering Sea. The commander squid is a very common commercial object, the byproducts of which constitute about 52% of the total animal’s weight [18]. These byproducts, which are usually considered waste, can be used to obtain biologically active substances including docosahexaenoic acid, which is necessary for the preparation of *N*-docosahexanoylethanolamine. Total lipids content in the commander squid digestive gland is about 20.0–35.6%, comprising mainly triacylglycerols (TAG; 57.1–77.3%) and diacyl glyceryl ethers (DAGE; 12.9–14.6%) [19]. At the same time, the DHA concentration in the commander squid’s lipid fraction is about 36%, which significantly exceeds the content of this polyunsaturated fatty acid in other organisms [20]. Thus, obtaining of DHA from squid byproducts is undoubtedly a profitable enterprise. 

## 2. Results

### 2.1. In Vitro Studies 

**Figure 2:**
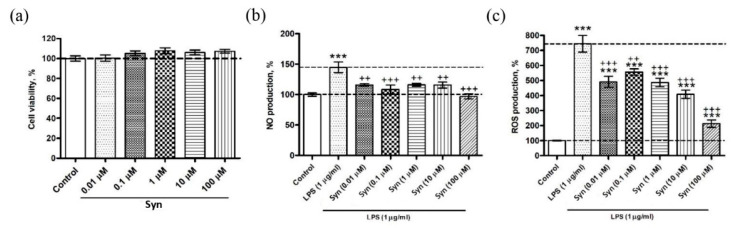
The in vitro study of the synaptamide (Syn) biological activity using the microglial cell line (SIM-A9). (**a**) The effect of synaptamide on microglial cell viability. Data are the means ± SEM, *n* = 8 per group (number of wells), *p* < 0.05. (**b**) Inhibiting effect of synaptamide on lipopolysaccharide (LPS)-induced NO production. Data are the means ± SEM, *n* = 8 per group, *** *p* < 0.001, +++ *p* < 0.001, ++ *p* < 0.01. (**c**) Inhibiting effect of synaptamide on LPS-induced reactive oxygen species (ROS) production. Data are the means ± SEM, *n* = 8 per group, *** *p* < 0.001, +++ *p* < 0.001, ++ *p* < 0.01.

Cell viability was determined following synaptamide treatment for 48 h. We did not find a decrease in the viability of synaptamide-treated cells at any concentration used (Figure 2a). Determination of the cell NO production showed a significant increase in its levels of lipopolysaccharide (LPS)-treated cells. The addition of synaptamide at various concentrations to the cell culture prevented LPS-induced NO overproduction (F(6,48) = 9.56, *p* < 0.0001) (Figure 2b). The treatment of the cell culture with synaptamide reduced the production of reactive oxygen species (ROS) caused by LPS stimulation. The most effective in this regard was the dose of synaptamide 100 µM (F(6,49) = 46.66, *p* < 0.0001) (Figure 2c).

### 2.2. Behavioral effects of Synaptamide in Neuropathic Pain

#### 2.2.1. Thermal Hyperalgesia

**Figure 3:**
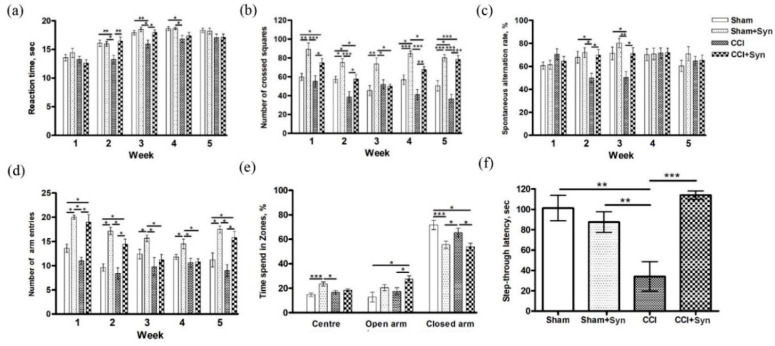
Effects of neuropathic pain and synaptamide treatment on animal behavior. (**a**) Hot allodynia: the moment of tail flick. (**b**) Locomotor activity: the number of crossed squares in the open-field test. (**c**) Working memory: spontaneous alternation rate in the Y-maze test, %. (**d**) Locomotor activity: the number of Y-maze arm entries. (**e**) The time spent in elevated plus-maze zones, %. (**f**) Long-term memory: step-through latency in the passive avoidance test, sec. Data are the means ± SEM, *n* = 10 (number of animals per group), * *p* < 0.05, ** *p* < 0.01, *** *p* < 0.001.

Data were analyzed using two-way ANOVA to assess the sizes of “treatment” (synaptamide treatment influence) and “surgery” (CCI or sham surgery) effects on the studied parameters. The study of thermal allodynia by a tail flick test showed the presence of hypersensitivity in the CCI group from two to four weeks after the surgery. At the same time, the synaptamide administration prevented the thermal hyperalgesia development in animals with neuropathic pain at weeks 2 (F (3,200) = 5.74, *p* = 0.017 for “treatment”; F (3,200) = 4.28, *p* = 0.044 for “surgery”) and 3 (F (3,200) = 5.86, *p* = 0.016 for “treatment”; F (3,200) = 5.54, *p* = 0.02 for “surgery”) after the surgery (Figure 3a).

#### 2.2.2. Locomotor Activity

An open-field study revealed that synaptamide treatment significantly induced locomotor activity in CCI- and sham-operated animals at week 1 (F (3,36) = 19.67, *p* = 0.0001 for “treatment”; F (3,36) = 2.98, *p* = 0.092 for “surgery”), week 2 (F (3,36) = 14.14, *p* = 0.001 for “treatment”; F (3,36) = 12.41, *p* = 0.001 for “surgery”), week 4 (F (3,36) = 38.79, *p* = 0.001 for “treatment”; F (3,36) = 14.35, *p* = 0.001 for “surgery”), and week 5 (F (3,36) = 67.98, *p* < 0.001 for “treatment”; F (3,36) = 4.05, *p* = 0.049 for “surgery”) after the surgery. Moreover, in synaptamide-treated CCI animals, the locomotor activity was significantly higher than that in vehicle-treated CCI animals at week 1 (F (3,36) = 8.25, *p* < 0.05), week 2 (F (3,36) = 9.42, *p* < 0.05), week 4 (F (3,36) = 18.55, *p* < 0.01), and week 5 (F (3,36) = 24.43, *p* < 0.001) (Figure 3b).

The Y-maze study of locomotor activity did not reveal a decrease in the number of entries in sham-operated animals with neuropathic pain. However, a significant increase in this indicator was found in both sham-operated animals and animals with neuropathic pain treated with synaptamide (*p* < 0.05) (Figure 3d).

#### 2.2.3. Anxiety

A study in the elevated plus maze did not reveal significant differences between the Sham and CCI groups. However, we found in the synaptamide-treated groups a significant decrease in the percentage of the time spent in the closed maze arms (F (3,36) = 24.88, *p* = 0.001 for “treatment”; F (3,36) = 3.18, *p* = 0.078 for “surgery”). At the same time, the synaptamide-treated sham-operated animals preferred to be in the center of the maze (F (3,36) = 12.59, *p* = 0.001 for “treatment”; F (3,36) = 1.13, *p* = 0.29 for “surgery”), and the animals of the CCI + Syn group spent more time exploring the open arms compared to the other groups (F (3,36) = 8.011, *p* = 0.006 for “treatment”; F (3,36) = 3.72, *p* = 0.049 for “surgery”) (Figure 3e).

#### 2.2.4. Working Memory

A study in the Y-maze showed a decrease in working memory in animals with neuropathic pain at weeks 2 (67.98, 5.38 in “Sham” vs. 49.99, 4.10 in “CCI”, F (3,36) = 4.90, *p* < 0.05) and 3 (71.38, 6.04 in “Sham” vs. 50.35, 5.49 in “CCI”, F (3,36) = 5.86, *p* < 0.05) following the surgery. In animals treated with synaptamide, the working memory indices did not significantly differ from the Sham group between weeks 2 (F (3,36) = 5.80, *p* = 0.021 for “treatment”; F (3,36) = 4.20, *p* = 0.048 for “surgery”) and 3 (F (3,36) = 7.37, *p* = 0.011 for “treatment”; F (3,36) = 7.61, *p* = 0.01 for “surgery”) after the surgery (Figure 3c).

#### 2.2.5. Long-Term Memory

Passive avoidance test with a 24-h retention interval revealed a decrease in the long-term memory in the CCI group compared to the Sham group. In the CCI + Syn group, there were no significant differences with the Sham group (F (3,36) = 9.58, *p* = 0.004 for “treatment”; F (3,36) = 4.63, *p* = 0.048 for “surgery”) (Figure 3f).

### 2.3. Effects of Synaptamide on Neuropathic Pain-Mediated Changes in Microglia Activity and Hippocampal Neurogenesis

#### 2.3.1. Microglia Activity and Cytokines Production

**Figure 4:**
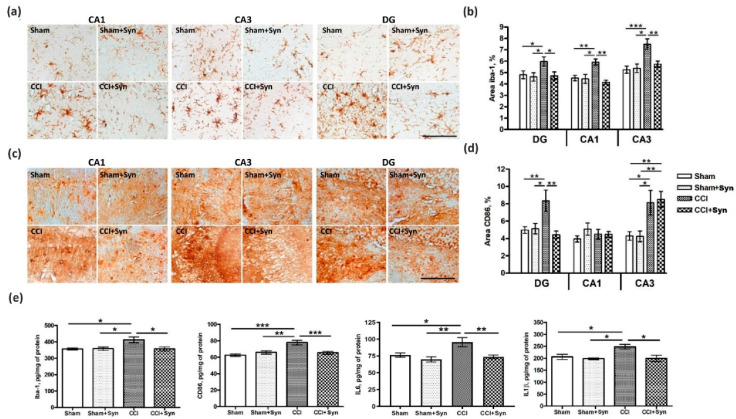
Effect of CCI and synaptamide treatment on hippocampal microglial activity. (**a**) Representative images of Iba-1 immunostaining. Scale bar: 100 μm. (**b**) Histogram demonstrating the % of Iba1^+^ staining within the CA1, CA3 and DG hippocampal regions after the surgery and synaptamide treatment. Mean ± SEM, *n* = 40 (number of slices), * *p* < 0.05, ** *p* < 0.01. (**c**) Representative images of immunostaining. Scale bar: 100 μm. (**d**) Histogram demonstrating the % of CD86^+^ staining within the CA1, CA3 and DG hippocampal regions after the surgery and synaptamide treatment. Mean ± SEM, *n* = 40 (number of slices), * *p* < 0.05, ** *p* < 0.01. (**e**) Effects of CCI and synaptamide treatment on hippocampal microglial marker and cytokine expression measured by ELISA; histograms of Iba-1, CD86, Il-1β, and Il-6 hippocampal expression. Mean ± SEM, *n* = 5 per group (number of animals), * *p* < 0.05, ** *p* < 0.01, *** *p* < 0.001.

The study of the synaptamide effect on microglial activity within the hippocampus was carried out by immunohistochemical staining for a microglial marker Iba-1 and a M1-specific microglial marker CD86. We found an increase in Iba-1-positive immunostaining for the neuropathic pain in the hippocampal dentate gyrus (F (3,156) = 5.47, *p* = 0.021 for “treatment”; F (3,156) = 5.93, *p* = 0.019 for “surgery”), CA1 (F (3,156) = 9.72, *p* = 0.002 for “treatment”; F (3,156) = 8.57, *p* = 0.004 for “surgery”) and CA3 regions (F (3,156) = 4.24, *p* = 0.04 for “treatment”; F (3,156) = 17.12, *p* < 0.001 for “surgery”). Moreover, the area of Iba-1-positive immunostaining in the group of synaptamide-treated animals with neuropathic pain did not significantly differ from that in the groups of sham-operated animals treated with synaptamide and vehicle (Figure 4a,b).

The study of CD86-immunopositive staining in the dentate gyrus revealed an increase in the CCI group, but in the CCI + Syn group no increase in CD86-immunopositive staining was observed (F (3,156) = 9.20, *p* = 0.003 for “treatment”; F (3,156) = 4.55, *p* = 0.036 for “surgery”). However, in the CA3 region, the administration of synaptamide did not prevent an increase in the CD86-immunopositive microglia area staining. The CD86 staining in the CCI + Syn group turned out to be at the level of the CCI group (F (3,156) = 0.032, *p* = 0.859 for “treatment”; F (3,156) = 20.05, *p* < 0.001 for “surgery”). In the CA1 region, changes in CD86 immunoreactivity were not observed with either pain or therapy (Figure 4c,d).

Immunohistochemical data on microglial activity were confirmed by data obtained using ELISA. It was shown that the Iba-1 concentration in the hippocampus of vehicle-treated animals with CCI was significantly increased in comparison with the Sham group. In synaptamide-treated rats with CCI, the concentration of Iba-1 was significantly lower than in the CCI group (F (3,36) = 4.30, *p* = 0.044 for “treatment”; F (3,36) = 4.52, *p* = 0.042 for “surgery”). A similar situation was observed for CD86 accumulation. In vehicle-treated animals with CCI, the number of CD86 was significantly higher than in the synaptamide-treated group of animals with CCI (F (3,36) = 4.71, *p* = 0.037 for “treatment”; F (3,36) = 12.50, *p* < 0.001 for “surgery”). ELISA of cytokines in the hippocampal tissue showed an increase in the pro-inflammatory cytokine IL-1β production in the CCI group compared to the CCI + Syn group (F (3,36) = 4.27, *p* = 0.048 for “treatment”; F (3,36) = 4.80, *p* = 0.035 for “surgery”). In addition, an increase was shown in the IL-6 concentration of the CCI group when compared with the CCI + Syn group (F (3,36) = 8.80, *p* = 0.006 for “treatment”; F (3,36) = 5.84, *p* = 0.022 for “surgery”) (Figure 4e). A study of the IL-10 production did not reveal significant differences between the groups (data not shown).

#### 2.3.2. Neurogenesis

**Figure 5:**
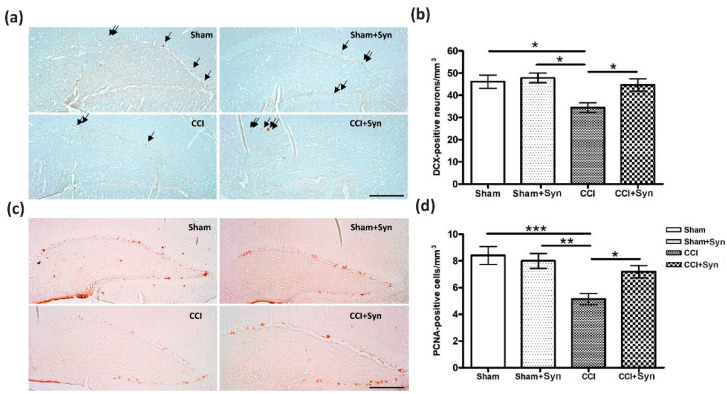
Effect of CCI and synaptamide treatment on hippocampal neurogenesis. (**a**) Representative images of proliferating cell nuclear antigen (PCNA^+^) staining. Scale bar: 100 μm. (**b**) Histogram showing the changes in the number of PCNA^+^ cells in the dentate gyrus subgranular zone (DG SGZ). (**c**) Representative images of doublecortin^+^ staining. Scale bar: 100 μm. (**d**) Histogram showing the changes in the number of immature neurons (by doublecortin) in the DG SGZ. Mean ± SEM, *n* = 20 (number of slices per group), * *p* < 0.05, ** *p* < 0.01, *** *p* < 0.001.

The study of the hippocampal neurogenesis processes was carried out by immunohistochemical detection of proliferating cell nuclear antigen (PCNA) and doublecortin (the marker of newly formed neurons). When calculating the number of PCNA- and doublecortin-immunopositive cells, only cells located strictly in the dentate gyrus subgranular zone (DG SGZ) of the hippocampus were considered. We found that in the vehicle-treated CCI animals the number of PCNA-immunopositive neurons was significantly reduced compared to the Sham and Sham + Syn groups (F (3,76) = 7.38, *p* = 0.0006), while in the CCI + Syn group, the average number of immunopositive neurons was significantly higher than in the CCI group (F (3,76) = 4.24, *p* = 0.048 for “treatment”; F (3,76) = 13.70, *p* < 0.001 for “surgery”) (Figure 5a,b). A similar result was observed when analyzing the immunohistochemical detection of doublecortin. We found a decrease in the doublecortin-positive neuron density in the DG SGZ in rats with neuropathic. In the CCI + Syn group, the average density of doublecortin-positive neurons was significantly higher than in the CCI group (F (3,76) = 4.69, *p* = 0.048 for “treatment”; F (3,76) = 5.63, *p* = 0.019 for “surgery”) (Figure 5c,d).

### 2.4. Effects of Synaptamide on Neuropathic Pain-Mediated Changes in Brain Lipid Composition

**Figure 6:**
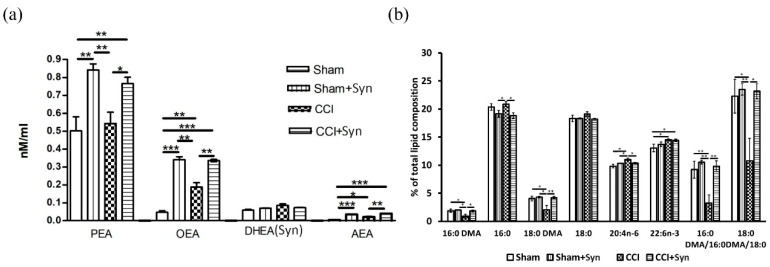
The results of the whole mouse brain LC-MS lipid analysis after CCI and synaptamide treatment. (**a**) *N*-acylethanolamines levels are expressed as nM/mL. (**b**) Brain content of the main fatty acids and DMA (% of total fatty acids and DMA). Mean ± SEM, *n* = 5 (number of animals per group), * *p* < 0.05, ** *p* < 0.01, *** *p* < 0.001.

Lipid analysis revealed an increase in the palmitoyl ethanolamide (PEA) concentration in the brains of synaptamide-treated animals both sham-operated and with neurotrauma (F (3,16) = 24.94, *p* = 0.0001 for “treatment”). In addition, an increase in the oleoyl ethanolamide (OEA) concentration was observed both in the CCI group compared to the Sham group (0.04 ± 0.01 in Sham vs. 0.19 ± 0.04 in CCI, p < 0.01) and in both synaptamide-treated groups (0.33 ± 0.01 in “CCI + Syn” and 0.34 ± 0.03 in “Sham + Syn”) (F (3,16) = 73.82, *p* = 0.0001 for “treatment”; F (3.16) = 6.88, *p* = 0.021 for “surgery”). We found the effect of both trauma and treatment on the arachidonyl ethanolamide (AEA) brain concentration (F (3,16) = 9.66, *p* = 0.008 for “surgery”; F (3.16) = 46.93, *p* < 0.001 for “Treatment”). An increase in AEA concentration was found in the CCI group when compared to the Sham group (0.0039 ± 0.001 in Sham vs. 0.021 ± 0.005 in CCI, *p* < 0.001). Moreover, in the synaptamide-treated groups, the increase of synaptamide concentration was even more pronounced: 0.04 ± 0.003 in “CCI + Syn" vs. 0.03 ± 0.002 in “Sham + Syn”. Interestingly, when synaptamide was administered to rats for five weeks, the brain lipid concentration did not increase significantly (Figure 6a).

In addition, we identified the effect of CCI and synaptamide therapy on the fatty acid composition within the rats’ brains. In particular, synaptamide administration was shown to prevent CCI-mediated decreases in 16:0 dimethyl acetal (DMA) levels (F (3,16) = 4.98, *p* = 0.037 for “treatment”; F (3.16) = 5.37, *p* = 0.044 for “surgery”) and 18:0 DMA (F (3,16) = 9.45, *p* = 0.018 for “treatment”; F (3.16) = 7.32, *p* = 0.009 for “surgery”). Neurotrauma led to an increase in the arachidonic acid (ARA) (F (3.16) = 8.22, *p* = 0.025 for “surgery”) and docosahexaenoic acid (DHA) (F (3.16) = 11.02, *p* = 0.006 for “surgery”) brain concentrations; however, treatment did not significantly affect PUFA composition. At the same time, synaptamide administration prevented CCI-induced decrease in the content of plasmalogen indirect markers 1,1-dimethoxyhexadecane (DMA 16: 0)/palmitate (C16: 0) (F (3,16) = 13.24, *p* = 0.003 for “treatment”; F (3.16) = 9.56, *p* = 0.009 for “surgery”) and 1,1-dimethoxyhexadecane (DMA 18:0)/octadecanoate (C18:0) (F (3,16) = 6.27, *p* = 0.028 for “treatment”; F (3,16) = 8.28, *p* = 0.014 for “surgery”) (Figure 6b).

## 3. Discussion

The present study aims to evaluate the effect of N-docosahexaenoylethanolamine (synaptamide) on neuroinflammatory response within the hippocampus during neuropathic pain. We investigated the effect of synaptamide on neuropathic pain severity, hippocampal-dependent memory, microglial activity, and hippocampal neurogenesis in a rat CCI model. Pain response in animals was evaluated using the tail-flick test by observing reactions to heat. We found that the greatest hyperalgesic response in the CCI group was observed two to three weeks post-surgery. It is worth noting that in the synaptamide-treated animals with ligated nerves, the hyperalgesic response was at the level of the sham-operated group, indicating synaptamide analgesic activity. For almost the entire observation period, there was no dramatic locomotor activity decrease in the group of nerve-ligated synaptamide-treated animals when compared against the CCI group. Interestingly, the group of sham-operated synaptamide-treated animals manifested a significant locomotor activity increase compared to the Sham group throughout the entire observation period. A similar result was observed when studying locomotor activity using the Y-maze. In addition, behavioral testing revealed hippocampus-dependent memory impairment in rats with a ligated sciatic nerve. In addition, we found that synaptamide prevented the impairment of the working spatial memory that occurred two to three weeks post-surgery in nerve-ligated vehicle-treated rats. A passive avoidance test revealed that synaptamide treatment also prevented context-dependent long-term memory impairment in rats with neuropathic pain. In addition, we found a decrease in the time spent in the elevated plus maze closed arms in the synaptamide-treated groups, which indicated the anxiolytic activity of the compound. Further immunohistochemical and biochemical studies shed light on some mechanisms underlying the observed phenomena, and observed behavioral effects may have been due to various factors, although the analgesic and cognitive effects were potentially based on increased brain anandamide concentration in synaptamide-treated rats. Since it was previously known that synaptamide does not have a direct effect on CB1 receptors, the analgesic activity of synaptamide cannot be due to their stimulation. This is confirmed in our study by the fact that the synaptamide administration increased locomotor activity, and it was previously proven that stimulation of CB1 receptors in the brain reduces mobility [21,22]. This is consistent with our results, according to which we observed an increase in locomotor activity in sham-operated synaptamide-treated rats, although lipid analysis revealed a significant increase in the anandamide brain concentration in synaptamide-treated rats, which can still stimulate CB1 receptors and have analgesic effects [23]. Moreover, in our study, synaptamide treatment for five weeks resulted in a significant brain accumulation of *N*-acylethanolamines (NAEs) PEA and OEA, but there was no significant increase in synaptamide content. This may indicate the main role of synaptamide as a source of precursors for the *N*-acylethanolamines synthesis, since most of the synaptamide entering the body is most likely hydrolyzed to form DHA and ethanolamine under the influence of fatty acid amide hydrolase (FAAH) [13]. Synaptamide hydrolysis products are involved in the phosphatidylethanolamine and *N*-acylphosphatidylethanolamine biosynthesis, which are the precursor of *N*-acylethanolamides. In addition, increasing synaptamide concentration can inhibit FAAH activity against other NAEs due to competitive displacement from binding to the substrate [24]. For example, in a study by Bisogno et al., 1999 [25], synaptamide inhibited AEA hydrolysis, although to a lesser extent than AEA itself. The observed synaptamide anti-inflammatory effect may also be based on an increase in the brain PEA content, which is known to reduce the intensity of neuroinflammation [26]. The anti-inflammatory activity of synaptamide was also shown in our experiments on a microglial cell culture, where the synaptamide treatment in the concentration ranged from 0.1 to 100 μM, demonstrating a decrease in ROS and NO production. The effectiveness of synaptamide in cell culture suggests that it probably acts through membrane receptors. The mechanisms of synaptamide anti-inflammatory activity were earlier studied in in vitro experiments where there was a decrease in NO production in the BV2 line of microgliocytes due to activation of the cAMP/PKA signaling pathway and inhibition of NF-κB activation [17].

In addition, recent data have demonstrated the role of GPR110 as the functional receptor of synaptamide [27]. GPR110, located on the membranes of microglial cells, transmits anti-inflammatory signals by increasing the level of cAMP, a regulator of innate and adaptive immune responses (Raker et al., 2016) [28]. The GPR110 receptor is located on peripheral and central innate immune cells, including brain microglia. Activation of this receptor by synaptamide attenuates microglial activity and causes pro-inflammatory cytokine production decrease [27], which we observed in our study. Our results demonstrate that synaptamide reversed hippocampal microglia activation in rats with neuropathic pain. In addition, the elevated hippocampal production of pro-inflammatory cytokines IL-1β and IL-6 was not observed in the synaptamide-treated rats. However, activity through intracellular receptors during the penetration of synaptamide into the cell should not be ruled out. Since it has been proven that the main activity of synaptamide is realized through the GPR110 receptor, this receptor can serve as a target for pharmacological modifications of neuroinflammatory processes, as well as for studying the biological effects of synaptamide. Moreover, it has been shown that while blocking receptor binding to synaptamide or GPR110 gene knockout eliminates synaptamide-induced biological activity, overexpression of GPR110 enhances it. In GPR110 knockout mice, recognition and spatial memory are significantly impaired [29]. The known DHA anti-neuroinflammatory and neuroprotective activity probably occur precisely due to an increase in synaptamide synthesis. At the same time, impaired memory and cognitive functions associated with a decreased brain DHA level and caused by insufficient dietary intake of omega-3 fatty acids can be explained by a decrease in the synaptamide production [30].

Maintaining a normal level of plasmalogens (Pls) within the brain plays an important role in reducing microglia activity during the synaptamide administration. It is known that plasmalogens contain a saturated fatty acid with a vinyl ester bond at the sn-1 position, as well as a polyunsaturated fatty acid (e.g., arachidonic or docosahexaenoic acid) at the sn-2 position, which is released by Pls-selective phospholipase A2 (PLA2) [31]. In our study, neuroinflammation induced by peripheral neurotrauma decreased brain plasmalogen content as indicated by dimethyl acetal forms of 16:0 (hexadecanaldimethylacetal) and 18:0 (octadecanaldimethylacetal). This decrease may be due to the oxidative stress development caused by the neuroinflammation process, since the Pls-specific vinyl ester bond at the sn-1 position can be damaged by oxidants, including reactive oxygen/nitrogen species [32]. In addition, Pls-selective PLA2 (the activity of which is significantly increased under inflammatory conditions) can metabolize plasmalogens, reducing their content in the brain [31]. It has recently become known that pro-inflammatory stimuli, stress, and aging suppress the expression of Pls-synthesizing enzyme, Gnpat, which leads to a decrease in Pls levels [33]. We did not observe a decrease in the plasmalogen levels in synaptamide-treated rats, which, on the one hand may be a consequence of NAE anti-inflammatory activity, or on the other hand could be a result of anti-inflammatory activity. At the same time, we also observed an increase in the total DHA content in both vehicle- and synaptamide-treated animals with peripheral neurotrauma. According to the ANOVA results, the main effect of the total DHA content in the brain was exerted by the presence/absence of trauma. This could mean that the release of DHA during synaptamide hydrolysis is not the main mechanism of the synaptamide anti-inflammatory activity, a fact that is supported by the observation of much lower DHA biological activity compared to synaptamide [16]. In vitro experiments showed that synaptamide activity is not blocked by cyclooxygenase and lipoxygenase, but decreases as a result of FAAH, which also confirms the hypothesis that synaptamide is the main metabolite that determines DHA neuroprotective activity [17]. In vivo studies using radioactive labels have shown that synaptamide accumulates to a greater extent in the brain than DHA after exogenous intake [34], which also explains the more pronounced activity. In our study, synaptamide administration prevented working spatial and long-term memory impairment in rats with neuropathic pain syndrome. Given that microglia activation and the pro-inflammatory cytokine production adversely affect hippocampus-dependent memory, it is most likely the synaptamide anti-inflammatory activity that underlies the observed effects. In addition, pronounced anti-inflammatory activity plays an important role in maintaining a normal level of hippocampal neurogenesis, which is impaired by neuropathic pain. Chronic neuropathic pain contributes to long-term behavioral adaptation associated with neuro-inflammatory signaling and impaired neuroplasticity. It recently became known that peripheral nervous system damage leads to impaired neuronal plasticity and changes in the activity of microglia and astrocytes, which, in turn, leads to an imbalance in cytokine production and to central sensitization [35]. In our study, pain-induced neuroinflammation disrupted hippocampal neurogenesis and contributed to the cognitive and affective deficits. The synaptamide administration for five weeks allowed us to maintain a normal level of proliferative activity in the DG SGZ.

We suggest that the observed pharmacological effects are largely due to the pronounced synaptamide anti-inflammatory activity. Pro-inflammatory cytokines are known to inhibit neurogenesis, while anti-inflammatory ones, on the contrary, stimulate the proliferation of hippocampal neurons and ensure their survival [36,37,38,39]. For instance, a decrease in neuronal proliferative activity within the hippocampus has been observed in transgenic mice with increased astrocyte IL-6 production [40]. And if the working memory impairment in neuropathic pain can be a consequence of neuroinflammation [41], then the long-term memory associative emotional learning deficiency that we identified in the passive avoidance test is probably the result of impaired hippocampal neurogenesis. We make such a conclusion based on studies confirming the involvement of hippocampal neurogenesis impairment in the development of hippocampus-dependent context learning disorders [42,43,44]. The hippocampus-specific behavioral deficit in context extinction [4] confirms the involvement of the hippocampal DG in pain signal processing and transmission. According to our data, synaptamide administration both prevented the violation of long-term hippocampus-dependent context learning and reduced the activity of CD86^+^ (pro-inflammatory M1) microglia in the DG. This suggests a connection between microglial activation in the DG, decreases in hippocampal neurogenesis, and impaired hippocampus-dependent memory. At the same time, we did not observe an increase in CD86+ microglia activity in the CA1 hippocampal area five weeks post-surgery, which may explain the spatial working memory recovery, since the functioning of this memory type is associated with the CA1 region [45,46]. Neuropathic pain-derived impaired working memory and the underlying morphological changes in the dendritic tree of CA1 pyramidal neurons are probably associated with axonal projections from layer III of the medial entorhinal cortex to the CA1 hippocampus region. Entrances from the third layer of the entorhinal cortex into the hippocampus are known to play an important role in the spatial working memory tasks [47,48]. This entry pathway, as well as axons extending from layer II of the entorhinal cortex to the DG dendrites, are the major input pathway of pain information to the hippocampus [5]. The increased Iba-1+ immunostaining in the CA1 region may indirectly indicate the activation of anti-inflammatory M2 microglia, secreting neuroprotective factors and contributing to the normal functioning of neurons in this area, leading to the working memory recovery in animals with a ligated sciatic nerve. In addition, the hippocampal CA1 region is known to be involved in anxiety-like behavior by altering the expression of GluR1 in response to various external stimuli [49]. The absence of increased anxiety in the elevated plus maze in synaptamide-treated animals in our experiments also emphasizes the relationship between the neuroinflammation processes within the CA1 area and their functional activity, indicating the anxiolytic activity of synaptamide. At the same time, we observed an increase in CD86+ immunostaining in the CA3 hippocampal region, while synaptamide administration did not reduce the activity of pro-inflammatory microglia in this area five weeks post-surgery. Given that the CA3 region is the most susceptible to changes under various stressful conditions [50,51], activation of microglia may in this case be not only a consequence of pain pathology, but also a consequence of chronic stress associated with hypothalamo-pituitary-adrenal (HPA) axis activation and the development of a maladaptive response [52]. In this case, the administration of synaptamide reduces the intensity of these changes, preventing them from leading to fatal neuronal dendritic tree structural abnormalities and the impairment of cognitive processes.

## 4. Materials and Methods 

### 4.1. Animals and Surgery

Male Wistar rats (250 ± 10 g, three months old) were used. The rats were obtained from the National Scientific Center of Marine Biology, Far Eastern Branch of the Russian Academy of Sciences, Vladivostok, Russia. The animals were housed two to three per cage with ad lib access to chow and water in a 12-h light/dark cycle. Temperature (23 ± 2 °C) and humidity (55 ± 15%) were constant. All experimental procedures were approved by the Animal Ethics Committee at the National Scientific Center of Marine Biology, Far Eastern Branch, Russian Academy of Sciences (No. 1/2020), according to the international regulations of the European Directive 2010/63/EU and ethical guidelines for the study of experimental pain in conscious animals by the International Association of the Study of Pain. The induction of neuropathic pain was performed using a model of sciatic nerve chronic constriction injury (CCI) [53]. Animals were anesthetized with isoflurane using rodent anesthesia vaporizer (VetFlo, Kent Scientific Corporation, Torrington, CT, USA). After the animal has been anesthetized, the right sciatic nerve was exposed and the three ligatures were placed closer to trifurcation with 1 mm between the ligatures (silk, Ethicon, Somerville, NJ, USA). The ligatures were slightly tightened until a slight twitching of the limb appeared. 

### 4.2. N-Docosahexaenoylethanolamine Preparation

Synaptamide was obtained from the processed products of Far-Eastern squid (*Berryteuthis magister*) caught in the Bering Sea. The concentrate of polyunsaturated fatty acid was obtained according to the method of Ermolenko et al. [54]. Further, the polyunsaturated fatty acid concentrate was converted to ethyl esters then treated with ethanolamine to obtain ethanolamides of fatty acids. HPLC of polyunsaturated fatty acid ethanolamides was performed on a Shimadzu LC-8A chromatograph (Shimadzu, Kyoto, Japan) with UV/VIS SPD-20A (205 nm). Separation was performed on a preparative reverse-phase column Supelco Discovery HS C-18 (Sigma Aldrich, Bellefonte, PA, USA); 10-µm particle size, 250 × 50 mm i.d. Isocratic elution with an ethanol/water (70:30, *v*/*v*) system was used. The elution rate was 50 mL/min. Fractions containing ethanolamide of DHA (synaptamide) were collected, evaporated under vacuum, and analyzed by gas chromatography (GC) and GC mass spectrometry (GC-MS). Synaptamide was a light-yellow oil fluid with an unexpressed smell at room temperature. The purity was 99.4%.

GC and GC-MS techniques were used to determine the trimethylsilyl derivative (TMS-NAE) compositions of fatty acid ethanolamide. For TMS-NAE preparation, 50 μL of *N*,*O*-bis(trimethylsilyl)trifluoroacetamide (BSTFA) were added to 1 mg of fatty acid ethanolamides followed by heating up to 60 °C for 1 h under argon. Then, 1 mL of hexane was added, and 1 μL of each silylated fraction was injected into the GC system. For TMS-NAE composition determination, we used a Shimadzu GC-2010 chromatograph with a capillary column Supelco SL-5 ms 30 m × 0.25 mm i.d. (Sigma Aldrich, Bellefonte, PA, USA) and flame ionization detector (Shimadzu, Kyoto, Japan). The mixture components were separated under specific conditions: (1) initial temperature of 180 °C; (2) heating rate of 2 °C/min to 260 °C; and (3) temperature was maintained for 35 min. The injector and detector temperatures were equal and amounted to 260 °C. For TMS-NAE structure identification, GC-MS was used. Electronic impact spectra were recorded using the Shimadzu TQ-8040 (Shimadzu, Kyoto, Japan) instrument with a Supelco SL-5 ms (Sigma Aldrich, Bellefonte, PA, USA) column at 70 eV. The same temperature conditions were used as during gas chromatography.

### 4.3. Treatment

Synaptamide was administered to the rats subcutaneously as a water emulsion. The emulsion was prepared by mixing synaptamide with water to obtain a final concentration of 25 mg/mL with constant shaking using a Multi-Vortex shaker (V-32, Biosan, Riga, Latvia). The resulting emulsion was injected subcutaneously in rats in such an amount to provide a dose of 10 mg/kg of synaptamide. The period of administration was five weeks daily from the day the surgery was performed. The animals (*n* = 60) were randomly divided into four groups: (1) the “Sham” group (*n* = 15), comprising vehicle (water)-treated sham-operated animals; (2) the “Sham + Syn” group (*n* = 15), comprising synaptamide-treated sham-operated animals. (3) the “CCI” group (*n* = 15), comprising vehicle-treated animals subjected to sciatic nerve constriction injury; and (4) the “CCI + Syn” group (*n* = 15), comprising synaptamide-treated animals subjected to sciatic nerve constriction injury. The rats were sacrificed 35 days after the surgery on the day of the final synaptamide or vehicle administration.

### 4.4. Cell Culture

SIM-A9 murine microglia cell (CRL-3265, ATCC’s collections, Manassas, VA, USA) line was obtained from the American Type Culture Collection. The cells were cultured in Dulbecco’s Modified Eagle’s medium (DMEM) containing 10% fetal bovine serum (FBS) at 37 ° C in a humidified incubator (MCO-18AIC, Sanyo, Osaka, Japan) atmosphere with 5% CO_2_.

### 4.5. MTS Assay

Synaptamide cytotoxic activity was determined using MTS (CellTiter 96 AQ_ueous_ One Solution Cell Proliferation Assay, Promega, Madison WI, USA) following the manufacturer’s instructions. Briefly, SIM-A9 murine microglia were plated into 96-well microplates (1 × 103 cells/well) and incubated at 37 °C with 5% CO_2_ for 1 h. After adhesion, cells were incubated at 37 °C, 5% CO_2_ with synaptamide solution (0.01, 0.1, 1, 10, 100 μM) for 24 h, or with the culture medium (control cells). Then, 10 μl of the reagent was added to each well. The plate was incubated for 2 h at 37 °C, and the absorbance was determined at 490 nm with an iMark microplate absorbance reader (Bio-Rad, Hercules, CA, USA). Cytotoxic activity was expressed as the percent of cell viability.

### 4.6. Reactive Oxygen Species Determination

SIM-A9 murine microglia were plated into 96-well microplates (1 × 103 cells/well) and incubated at 37 °C with 5% CO_2_ for 1 h. After adhesion, cells were incubated at 37 °C, 5% CO_2_ with LPS solution (1 μg/mL) and synaptamide (0.01, 0.1, 1, 10, 100 μM) for 24 h. Cells incubated with LPS solution (1 μg/mL) were used as a positive control; cells treated with culture medium were used as a negative control. To determine the production of reactive oxygen species, 20 μL of 2,7-dichlorodihydrofluorescein diacetate solution (Molecular Probes, Eugene, OR, USA) was added to the wells with cells to obtain a final concentration of 10 mM. Then, the microplate was incubated for 10 min at 37 °C (Ivanchina et al., 2015). A PHERAstar FS plate spectrophotometer (BMG Labtech, Ortenberg, Germany) was used to measure the fluorescence intensity of dichlorofluorescein. Measurements were performed at wavelengths λex = 485 nm and λem = 518 nm. Data processing was carried out using the MARS Data Analysis v. 3.01R2 software (BMG Labtech, Ortenberg, Germany).

### 4.7. Nitric Oxide Determination

To study NO production by cells, a reaction with 10 μM DAF-FM diacetate (4-Amino-5-Methylamino-2′,7′-Difluorofluorescein Diacetate, Thermo Fisher Scientific, Waltham, MA, USA) was performed for 40 min at 37 °C. Then, the cells were washed three times in PBS (pH 7.4) and placed into the wells of the plate containing 200 μL of PBS. Fluorescence was recorded at wavelengths λex = 460 nm and λem = 524 nm using a PHERAstar FS plate reader (BMG Labtech GmbH, Ortenberg, Germany). Cells treated with a solution of LPS (*E. coli* O111: B4, Sigma-Aldrich, St. Louis, MO, USA) at a concentration of 1 μg/mL were used as a positive control. Each in vitro experiment was carried out independently at least three times.

### 4.8. Behavioral Tests

#### 4.8.1. Tail Flick Test

To assess the antinociceptive effect of synaptamide, the Tail Flick Analgesia Meter and the restrainer LE5022 (Panlab, Barcelona, Spain) were used. For this, the tail was placed on a heating element where the heating point was located at a distance of 5–8 cm from the tip of the tail. The cut-off point was set at 10 s for a tail response sufficient to interrupt tissue damage. The reaction time of the animal, expressed by the movement of its tail, was recorded.

#### 4.8.2. Elevated Plus Maze

Animal anxiety studies were performed using an elevated plus maze (Panlab, Barcelona, Spain). The study was based on the tendency to avoid open spaces with increased anxiety. The maze was a device consisting of two open arms (30 × 5 cm, 0.25-cm-high walls) and two closed arms (30 × 5 cm, 15-cm-high walls), emerging from the central platform (5 × 5 cm), located opposite to one another. The animal was placed in the center of the maze facing the open arm and left in the apparatus for 5 min, with behavior recorded using a video camera located above the maze. The times spent in the open and closed arms and in the central zone were recorded automatically.

#### 4.8.3. Spontaneous Locomotor Activity

Locomotor activity was assessed using open field testing. Each animal was placed in the center of a square plexiglass arena (width: 50 cm; height: 40 cm) for 5 min. The arena was divided into 25 squares. The behavior of the animal was recorded using a video camera placed over the device, and the number of crossed squares was counted.

#### 4.8.4. Working Memory

The assessment of working memory in rats was carried out using the spontaneous alternation test in the Y-maze. The Y-maze was an apparatus made of opaque acrylic glass with three equal arms (50 cm long, 32 cm high, and 16 cm wide). Each rat was placed in the center of the maze for 5 min to move freely and explore the surrounding space. Entering the arm was counted when the animal entered the arm with all four paws. To calculate the spontaneous alternations rate, the total number of entries (N) and the number of "correct" triplets (M, the sequential choice of each of the three branches without repeated entries) was estimated. The alternations rate was calculated using the following formula: R (%) = M × 100/(N − 2).

In addition to working memory, the animal’s locomotor activity was assessed using the Y-maze testing. To this end, the total number of entries into the arms of the maze was recorded for each animal.

#### 4.8.5. Passive Avoidance Test

The passive avoidance test has been used to assess the effects of chronic neuropathic pain and synaptamide treatment on long-term memory [55]. The test apparatus consisted of light and dark chambers separated by a sliding door. During the training phase, the rats were placed in the light chamber and allowed to move freely for 60 s before opening the sliding door. When the animal entered the dark chamber, the door was closed, and after 2 s an electric foot-shock was produced (0.3 mA, 2 s). The test stage was carried out 24 h after training, but without electric foot-shock. To assess long-term memory, we measured the delay before the animals entered the dark chamber (step-through latency).

### 4.9. Immunohistochemistry

The samples for subsequent histological analysis were collected on the 35th day post-surgery. Inhalation anesthesia was performed using isoflurane supplied with a rodent anesthesia vaporizer (VetFlo, Kent Scientific Corporation, Torrington, CT, USA). Transcardial perfusion was carried out using 100 mL of saline at a temperature of about 4 °C, (pH 7.2). The rat brains were extracted, divided into two hemispheres, and fixed for 12 h at 4 °C in buffered 4% paraformaldehyde (PFA). Then, the tissue samples were embedded in paraffin blocks and sagittal sections with a thickness of 10 μm were made using a Leica rotary microtome (RM 2245; Leica Biosystems, Buffalo Grove, IL, USA). After deparaffinization, the sections were incubated in 3% hydrogen peroxide to block endogenous peroxidase activity. After three washes with 0.1 M phosphate buffer (pH 7.2), the sections were placed in blocking solution for 60 min, consisting of 2% bovine serum albumin solution (sc-2323; Santa Cruz Biotechnology, Santa Cruz, CA, USA) and 0.25% Triton X-100 (Sigma, St. Louis, MO, USA). Slides with sections were incubated with primary antibodies (rabbit polyclonal antibodies against Iba-1, 1:500, ab108539; rabbit monoclonal antibodies against CD86, 1:1000, ab53004; rabbit polyclonal antibodies against doublecortin, 1:500, ab18723; mouse monoclonal antibodies against PCNA, 1:500, ab29; Abcam, Cambridge, MA, USA) in a humidified chamber at 4 °C for 24 h. After three washes with 0.1 M phosphate buffer (pH 7.2), the sections were incubated with secondary antibodies (anti-rabbit, conjugated with horseradish peroxidase, 1:100, PI-1000; anti-mouse, conjugated with horseradish peroxidase, 1:100, PI-2000, Vector Laboratories, Burlingame, CA, USA) for 45 min. After washing with 0.1 M phosphate buffer (pH 7.2), the sections were treated with a chromogen solution (DAB Plus; Thermo Fisher Scientific, Waltham, MA, USA) until the desired staining was obtained. The sections were then washed with 0.1 M phosphate buffer (pH 7.2), dehydrated, and mounted in Dako’s toluene-free mounting medium (CS705; Dako, Carpinteria, CA, USA).

### 4.10. Immunostaining

To assess microglial activity within the hippocampus, immunohistochemical staining was performed using rabbit anti-Iba-1 polyclonal antibodies (1:500, ab108539) and anti-CD86 rabbit monoclonal antibodies (1:1000, ab53004) (Abcam, Cambridge, MA, USA). To assess neurogenesis intensity, the number of newly formed neurons in the dentate gyrus (DG) subgranular zone (SGZ) was determined using anti-doublecortin antibodies (anti-DCX) (1:500, ab18723; Abcam, Cambridge, MA, USA). Proliferative activity in the DG SGZ was determined by immunohistochemical staining of the nuclear antigen of proliferating cells (PCNA) using mouse monoclonal anti-PCNA antibodies (1:500, ab29) (Abcam, Cambridge, MA, USA). Secondary antibodies conjugated to horseradish peroxidase (PI-1000, anti-rabbit; PI-2000, anti-mouse) were used according to the manufacturer’s recommendations (1:100; Vector Laboratories, Burlingame, CA, USA).

### 4.11. Image Analysis

The images were obtained using a Zeiss Axio Imager microscope equipped with an AxioCam 503 color video camera and AxioVision software (Carl Zeiss, Oberkochen, Germany). The images were saved as TIFF files with subsequent processing and analysis using ImageJ software (NIH, Bethesda, MD, USA). Every sixth slice was used to quantify DCX and PCNA. Immunopositive cells were counted only within the DG SGZ. Only whole cells containing visible nuclei were used in the calculations. The area of the dentate gyrus was multiplied by the slice thickness and expressed in mm^3^. The following formula was used to calculate the number of immunopositive cells per mm^3^: d = (10^6^xn)/(Sxl), where “d” means cell density, “n” is the number of immunopositive cells, “S” is SGZ area (μm^2^), l is the slice thickness, and 10^6^ is the conversion factor for μm^2^ to mm^2^.

Estimation of the Iba1- and CD86-specific stained area was performed using each sixth section. For this, the images were processed in the following way: image conversion to grayscale mode, background subtraction, and binarization. The area occupied by Iba-1 and CD86 immunopositive microglia was then calculated in the CA1, CA3, and DG regions. All measurements were performed by an operator who was blinded to the identity of the sections.

### 4.12. ELISA

To determine the Iba1, CD86, IL-6, and IL-1β protein concentrations within the hippocampus, an enzyme-linked immunosorbent assay (ELISA) was used. Rats were anesthetized with isoflurane using rodent anesthesia vaporizer (VetFlo, Kent Scientific Corporation, Torrington, CT, USA) and each hippocampus was quickly extracted, frozen in liquid nitrogen, and stored at a temperature of −70 °C. The following ELISA kits were used for quantification of Iba1 (NBP2-66675, NovusBio), CD86 (RAB0887-1KT, Sigma-Aldrich, St. Louis, MO, USA), IL-6 (ERA32RB, Invitrogen), and IL-1beta (BMS630, Invitrogen) according to the manufacturer’s instructions. For analysis, we used both right and left hippocampi. The homogenization buffer consisted of 100 mM Tris, pH 7.4, 150 mM NaCl, 1 mM EGTA, 1 mM EDTA, 1% Triton X-100, and 0.5% sodium deoxycholate, with a protease inhibitor cocktail (cOmplete, Sigma-Aldrich, St. Louis, MO, USA). The BCA Protein Assay Kit (Pierce, Rockford, IL, USA) was used for determining protein concentration. The optical density was measured in an iMark plate spectrophotometer (Bio-Rad, Hercules, CA, USA) at a wavelength of 450 nm. 

### 4.13. Quantitation of NAE in Brain Lipids

An additional cohort of 20 animals was taken for lipid analysis. For lipid analysis, animals were euthanized with isoflurane using rodent anesthesia vaporizer (VetFlo, Kent Scientific Corporation, Torrington, CT, USA) and each brain was quickly extracted, frozen in liquid nitrogen, and stored at of -70 °C until use. An internal standard 22:0-NAE in a concentration of 0.1 nmol was added to frozen rat brain followed by lipid extraction according to Bligh and Dyer’s method (1959) [56]. The final lipid residue was reconstituted in 1 mL of ice-cold CHCl3, purged with argon, and stored at −80 °C until liquid chromatography-mass spectrometry (LC-MS) was performed. 

NAE analysis by LC-MS was achieved by Ascentis C18 analytical column (2.1 mm × 100 mm × 3-micron, Supelco, Bellefonte, PA, USA) and LC-MS 8060 (Shimadzu, Kyoto, Japan) equipped with an atmospheric-pressure-heated electospray ionization source in positive polarity mode. The mixture was analyzed by multiple reaction monitoring (MRM). The ion source parameters were set at: interface temperature, 380 °C; desolvation line temperature, 250 °C; nebulizing gas (N2) flow, 3 L/min; drying gas (N2) flow, 3 L/min; and heating gas (dry air) flow, 17 L/min. Collision energy was optimized for each compound individually. The molecular ion and fragment for each compound measured were as follows: 348→62 for AEA, 372→62 for DHEA, 326→62 for OEA, 300→62 for PEA, 328→62 for SEA, and 384→62 for the internal standard (22:0-NAE). Quantification of each NAE in the tissue sample was achieved using LabSolution (Shimadzu) followed by comparison of the peak area with the internal standard peak area. Values are expressed as nmoL/mL. 

### 4.14. Determination of Fatty Acid and Dimethyl Acetals Composition

Fatty acid methyl esters (FAMEs) and dimethyl acetals (DMAs) from the liver lipids of rats were prepared according to the procedure of Carreau and Dubacq [57]. α,β-unsaturated ether in plasmalogen molecules was converted into a DMA of the corresponding aldehyde during transesterification, and the relative amount of plasmalogen was thus reflected in the ratio between 18:0 aldehyde and stearic acid, as well as by the ratio between 16:0 aldehyde and palmitic acid [58]. An analysis of FAMEs and DMAs was conducted by gas chromatography on a Shimadzu GC-2010 chromatograph (Shimadzu, Kyoto, Japan) with a flame-ionization detector and a capillary column of 30 m × 0.25 mm i.d. Supelcowax 10 (Bellefonte, PA, USA). The analysis was carried out under the following conditions: the temperature of column was 190 °C, and the temperature of the injector and detector amounted to 240 °C. Helium was used as a carrier gas. The peaks of the fatty acid methyl esters were identified by retention times of individual fatty acid esters in comparison with the authentic standards of their equivalent carbon length numbers. DMA identification was carried out via a comparison of retention times with standards of 16:0DMA and 18:0DMA (Sigma-Aldrich, St. Louis, MO, USA). GC-MS was used to confirm the FAME and DMA structures. Electronic impact spectra were recorded using a Shimadzu TQ-8040 instrument (Kyoto, Japan) with Supelco SL-5 ms (Bellefonte, PA, USA) column at 70 eV under specific conditions: (1) an initial temperature of 160 °C; (2) a heating rate of 2 °C/min to 240 °C; and (3) maintain the temperature for 35 min.

### 4.15. Statistical Analysis

The data are expressed as the means ± SEM. Normality of the data was evaluated with a Shapiro–Wilk test. The data obtained by the behavioral tests, immunohistochemistry, ELISA and lipid analysis were compared using two-way ANOVA followed by a post hoc Tukey’s multiple comparison test. We use a two-way ANOVA to estimate the size of each effect, namely synaptamide treatment (“treatment”) and sciatic nerve ligation (“surgery”). The data obtained by in vitro studies were subjected to one-way ANOVA followed by a post hoc Tukey’s multiple comparison test to spot differences between multiple samples. The differences were considered statistically significant when the *p* value was <0.05. All statistical tests were performed using Microsoft Excel software (Microsoft, Tulsa, OK, USA) and GraphPad prism 4 (GraphPad Software, San Diego, CA, USA).

## 5. Conclusions

Overall, the findings of this study demonstrate that synaptamide treatment prevented behavioral effects resulting from neuropathic pain in rats. It is likely that the observed beneficial effect on behavioral parameters was based on the powerful anti-inflammatory activity of synaptamide, confirmed through both in vitro and in vivo studies. The modulating effect of the drug on the brain lipid composition, namely, on the plasmalogens content, determined synaptamide anti-inflammatory and neuroprotective effects. In conclusion, we show here that synaptamide may have potential for use in the prevention or treatment of the cognitive and emotional effects of neuropathic pain.
